# Is there a social justice variant of South–South health cooperation?: a scoping and critical literature review

**DOI:** 10.1080/16549716.2019.1621007

**Published:** 2019-07-17

**Authors:** Anne-Emanuelle Birn, Carles Muntaner, Zabia Afzal, Mariajosé Aguilera

**Affiliations:** a Dalla Lana School of Public Health, University of Toronto, Toronto, Ontario, Canada; b Bloomberg School of Nursing, University of Toronto, Toronto, Ontario, Canada; c Graduate Program in Health Policy and Equity, York University, Toronto, Ontario, Canada; d Independent Researcher, Miami, Florida, USA

**Keywords:** South-South cooperation, social justice, global health, health equity, Cuba

## Abstract

**Background**: In recent decades, global health scholars and policymakers have highlighted the burgeoning role of South–South cooperation (SSC) in health, claiming it constitutes a more just and even-handed approach to health cooperation. But the assertion that SSC *inherently* challenges power asymmetries and pursues egalitarian agendas and forms of interaction merits interrogation. Here we explore a transformative, counter-hegemonic, solidarity-oriented form of SSC – social justice-oriented South–South cooperation (SJSSC) – as differentiated from other types of health aid.

**Objective**: The objectives of this scoping review are: 1) to determine what is known and discussed through peer-reviewed and grey literature about SJSSC in health; and 2) to identify the different features and principles of SJSSC. This review seeks to inform research agendas and identify implications for policy and practice around SJSSC.

**Methods**: We conducted a search for relevant peer-reviewed and grey literature in eight languages and screened abstracts that met inclusion criteria. We carried out a full-text review and data extraction on included pieces and conducted a thematic analysis identifying a set of repeated themes related to the features and principles of SJSSC.

**Results**: We identified 188 publications meeting our criteria. Through an iterative process, we developed two overarching categories: values and strategies. Each comprises four themes that allowed us to map the ideas and practices of SJSSC depicted in the literature. The values mapped are: an anti-hegemonic world view; equity-oriented and redistributive political values; egalitarian terms of cooperation; and reciprocity. The strategies encompass: solidarity-building; health justice approaches; mutual exchange and collective justice; and challenging interests of dominant classes in the health arena.

**Conclusion**: This review rectifies ungrounded claims about SSC by identifying and mapping the research literature on SJSSC and has relevance for the conceptualization, policy development, and practice of equitable health cooperation.

## Background

In recent decades, proponents and practitioners of ‘global health diplomacy’ [1] have highlighted the burgeoning role of South–South cooperation (SSC) in health – that is, ‘the exchange of expertise between actors (governments, organizations and individuals) in [low and middle-income countries – LMICs]. Through this model of cooperation, [these] countries help each other with knowledge, technical assistance, and/or investments’ [2]. Claims have been made by global health scholars and actors directly involved in SSC, that SSC contrasts with prevailing health aid approaches [3–8]. Some hold that SSC is more equal than North–South health cooperation by its very essence [9–11]. According to this argument, former colonies in the Global South that are aid recipients-turned-donors behave differently than their Northern counterparts that have been colonial powers: SSC purveyors are considered to be more respectful, to operate on a more level playing field, to share policy goals and co-create cooperation agendas, and to refrain from imposing conditionalities on aid [12–15].

But while settings that have emerged from colonial domination may be open to cooperation that is less patronizing and self-interested, the assertion that SSC *inherently* challenges power asymmetries and pursues egalitarian agendas and forms of interaction merits interrogation. We examine whether the literature reveals the existence of a truly transformative – social justice-oriented – approach to health cooperation among countries of the Global South that distinguishes it from other forms of cooperation.

We first seek both to define and differentiate this transformative approach from the generic form of SSC. We build upon a prior historical and theoretical exploration of the relationships between actors, the nature of cooperation activities and practices, and the diplomatic, political, and social dimensions of SSC experiences that generated a novel concept of social justice-oriented South–South cooperation (SJSSC) [16].

The framework we utilize for this scoping review similarly goes beyond the standard conceptualizations of global health diplomacy [17–20]. We have found the dominant international relations dichotomy between realism and liberalism – even as massaged by institutionalism and constructivism [21] and reconciled through Joseph Nye’s [22] influential ‘soft power’ notion – to be inadequate. Although various SSC players have taken up soft power’s evocation of health diplomacy as a form of persuasion rather than compulsion [23,24] – yielding convenient outcomes for all parties – we find this concept to be used in a contradictory fashion and to be glaringly apolitical. The highly useful concepts of ‘horizontal cooperation’ and ‘structural cooperation’ [25], meanwhile, are crafted in the context of Brazilian SSC and not necessarily applicable beyond.

Instead, we have drawn on neo-Marxist and other heterodox international relations theories [26], settling on proletarian internationalism [27] – based on values of societal equity and resisting dominant power structures – to be the most useful in explaining the nature of SJSSC and in averting the concern that the loose employment of language of social justice, solidarity, and shared policy agendas has become so prolific so as to have lost meaning. As such, political values around radical redistribution of resources and power, including via transnational reciprocity, anchor our theoretical framework.

Based on our preliminary conceptualization, SJSSC presents a distinct variant of SSC converging on a mix of the following features [16]:

- Egalitarian terms of cooperation (reciprocal and respectful of sovereignty) aimed at reducing power asymmetries
- Counter-hegemonic values challenging capitalist and neoliberal interests
- Transformative aims towards health equity and social rights
- Mutual and locally engaged agenda-setting and community-based approaches.

Drawing from these ideas, the purpose of this scoping and critical literature review [28,29] is to identify, map, and analyze the existing literature (including grey literature) on the little-explored topic of SJSSC. We also have two accompanying objectives: to further refine our conceptualization of SJSSC and to inform research agendas and draw out implications for policy and practice around SJSSC.

### Research questions

1. What is known and discussed through documented literature (academic, grey, and official government sources) about SSC in health with features oriented to social justice (what we posit to be SJSSC) published from 1992 to the present, internationally and specifically in relation to Latin America and Africa?
2. What specifically are the different features and principles that characterize SJSSC?

We aim to transcend the aforementioned essentializing assumptions (i.e. that cooperation between Global South countries necessarily entails egalitarian partnering and pursues shared progressive policy agendas) that characterize much of the current literature on SSC. Ultimately, we hope to enable SJSSC to become recognized as a distinguishable and crucial alternative to other forms of cooperation involving countries in the Global South.

## Methods

### Search strategy

We conducted a scoping review of peer-reviewed and grey literature on SSC based on Arksey and O’Malley’s [28] methodological framework for scoping reviews and recommendations identified by Levac, Colquhoun, and O’Brien [30]. We identified relevant search terms and followed a highly iterative process of grouping together related terms and categorizing them into distinct sets (Appendix 1). We then used Boolean operators to capture the most relevant content. While we tried to be consistent in usage across the various databases, adjustments to the search strategy were necessary due to differences in syntax rules.

We reviewed all abstracts in English, Spanish, Portuguese, Italian, French, Catalan, German, and Russian. However, depending on the database, our use of English-language search terms and a more limited set of Spanish and Portuguese terms necessarily circumscribed our access to material published in other languages. We carried out special Spanish and Portuguese searches (with fewer search terms) to ensure our capture of these materials.

We conducted the search in 12 bibliographic databases (Medline, Healthstar, Proquest [IBSS, PAIS International, FRANCIS], Scopus, JSTOR, Web of Science, LILACS, Bioline, Directory of Open Access Journals, Google Scholar) and seven additional journals representing scholarship from Sub-Saharan Africa, Latin America, and pan-Third World venues (Tanzania Medical Journal, ACP-EU Trade Newsletter Pambazuka, United Nations Research Institute for Social Development, Intellectual Property Watch, and African Journal of AIDS Research, Bandung: Journal of the Global South, and Revista Eletrônica de Comunicação, Informação & Inovação em Saúde).

We searched for relevant books, book chapters, and dissertations in Google Books, WorldCat, and ProQuest Dissertations & Theses. We also considered it necessary to include website grey literature (Appendix 2) because many experiences of SSC may not have been documented within scholarly sources for various reasons, including the inequitable access to academic venues experienced by some Global South researchers and barriers to publishing in Global North-based, often for-profit, publications [31,32].

We also explored ProQuest, Web of Science, and Scopus for literature dealing with SSC specifically involving Cuba, Brazil, Venezuela, Ecuador, and Bolivia. Expecting these Latin American countries to be driven by a more social justice-oriented ethos in their global health diplomacy given their political orientation for a significant proportion of the time frame covered, we wanted to make sure our preceding searches had not overlooked any important publications, including government health diplomacy reports, to glean how governments characterized their own SSC activities.

### Review process: inclusion and exclusion criteria

Authors ZA and MA reviewed titles and abstracts to identify relevance to South–South health cooperation. Authors AEB and CM conducted a second review and removed unsuitable items, such as conference announcements. Included pieces then underwent a full-text review by two co-authors, conducted separately by ZA, AEB, and CM. The publications selected for full-text review were examined in detail to discern whether they described instances of SJSSC in health, meeting the criteria for inclusion in the scoping review shown in Table 1.

**Table 1.** Inclusion/exclusion criteria.

Criterion	Explanation
(1) Subject relevance	Cooperation must involve a health component.
(2) Type of literature	Excluded conference and special issue announcements and repeats of heavily overlapping literature by the same author(s).
(3) Type of cooperation relevance (South–South)	Cooperation must entail one of the following: bilateral (South–South), trilateral (South–South–South), multilateral, regional, or triangular (SSC mediated or financed by United Nations [UN] agencies or developed, capitalist countries).
(4) Clear critical component	Must involve some form of equity-oriented or leftist cooperation (redistributive, anti-imperialist, social democratic, socialist, etc.) rooted in the politics of solidarity, not charity.

### Data extraction

In a preliminary analysis phase, we developed a range of potential SJSSC components to analyze based on our assessment of existing literature. Data were extracted from all included publications as per Table 2.

**Table 2.** Dominant elements for data extraction.

Type of Data Extracted	Categories
Actors involved in the cooperation	i.e. countries and agencies
Type of cooperation	Bilateral Regional Triangular (South–Southcooperation also involving UN agencies or high-income countries) Multilateral (e.g. India, Brazil, and South Africa [IBSA]; Brazil, Russia, India, China, and South Africa [BRICS]) Trilateral (South–South–South)
Health-related activities:	Training and education Primary health care (PHC) and human resources Pharmaceutical production and distribution Knowledge transfer/sharing and research Health care infrastructure and equipment Disease control Domestic health policy International policy negotiations Surgery and non-PHC treatment
Social justice-oriented features of collaboration (here our review terms derive from sociological understandings of the actors, relations, and context of historical and contemporary experiences of proletarian internationalism, also drawing from our preliminary SJSSC characterization above) [34].	Shared agenda-setting and values Responsiveness to local needs Equitable partnering Policy autonomy and objectives Reciprocity Restitution, reparations, social debt
Contextual factors	i.e. cultural/linguistic, economic, geopolitical considerations
Goals of cooperation	i.e. health equity and solidarity as explicit goals
Publication type	academic or grey literature

### Analytic approach

From the extracted data, we tallied estimates on the dominant elements (Table 2) in the literature in terms of actors, types of cooperation, health-related activities, social justice-oriented features of collaboration, language, and publication type. We collated our preliminary analysis notes on contextual factors and goals of cooperation from the literature and identified a set of repeated themes related to the features and principles of SJSSC, as per our research questions. The included publications were then divided among the authors for a mapping exercise whereby authors individually reviewed articles and extracted quotations and ideas that illustrated the common themes we had identified.

This iterative process of reviewing the literature and discussing overlapping themes with the team led us to refine our categorization of features and principles into two overarching categories: 1) values; and 2) strategies (Table 3). The themes under values and strategies were identified both deductively – based on broad concepts established *a priori*, derived from a preliminary critical review of the literature – and inductively, that is, emerging from a finer-grained inductive identification of ideas based on thematic analysis of the data [33]. Drawing on our mapping exercise, authors AEB and CM conducted a critical thematic analysis of values and strategies identified in the SJSSC literature by synthesizing the results and further culling the thematic components. They then analyzed how these features distinguish SJSSC from other forms of global health cooperation and diplomacy, as explored further in the discussion.

**Table 3.** Themes related to values and strategies.

V A L U E S	
Themes and components	Selected illustrative quotes
1. Anti-hegemonic worldview Revolutionary: anti-imperialist/anti-capitalist vision; anti-neoliberal ~ challenging free market model Challenging North–South asymmetries of power (including in cooperation) Advocating internationalism; proletarian solidarity	‘The result is that ALBA can be seen to represent a new paradigm that permits the creation of a counter-hegemonic, network-like set of relations dominated by values of fairness, social justice and solidarity’ [38, p. 99]. ‘The Cuban government has consistently promoted an internationalist foreign policy based upon a philosophy of proletarian solidarity’ [39, p. 69]. ‘The liberation ethic and unity in African [health] diplomacy are both an assertion of interests and also a defensive strategy against the power imbalances African countries face in global negotiations’ [40, p. 11].
2. Equity-oriented and redistributive political values (within national politics and translated into cooperation activities) Socialism of the South Social democracy (reflecting working class power) Social inclusion Participatory democracy	‘The correlation between the altruistic character of a domestic policy, such as health, and the corresponding dimension of solidarity expressed in foreign policy and translated into international cooperation’ [41, p. 133]. ‘Between countries with similar ideologies,’ cooperation engenders ‘the idea of social inclusion and the responsibility of governments with a great commitment to the population, to the people. This is central to the construction of new, more transparent and horizontal democracies, also enabling cooperation’ [42, p. 14].
3. Egalitarian terms of cooperation Horizontal terms of interchange; sincerity No conditionalities Responsiveness to local needs and context-relevant solutions (autochthonous to Global South) Humanitarianism (solidarity-oriented/altruistic)	‘The South-South cooperation that Uruguay carries out is based on principles of solidarity among peoples, horizontality, equity, respect for national sovereignty and non-conditionality’ [43, p. 83]. ‘Brazilian engagement in South-South cooperation [involves]: 1) Solidarity 2) Response to the demands of developing countries 3) Adaptation of the Brazilian experience to the local context 4) No conditionalities 5) No association with commercial interests 6) No interference in the domestic affairs of partner countries’ [44, p. 15].
4. Reciprocity Harmonized interests and policies Support for national autonomy and self-determination Respect for sovereignty (including via shared identities as former colonies) Unity, ubuntu, ‘liberation ethics’	China’s engagement in Africa is ‘guided by principles of non-interference with domestic affairs of a recipient country, mutual benefits in economic development and self-determination among partner countries’ [45, p. 9]. ‘Day-to-day learning, with real-world work experiences, is a process of collective, horizontal, and shared building, still a challenge for us all’ [46, p. 49]. ‘South Africa’s diplomatic activities also reflect the principles of state sovereignty, non-interventionism and resistance to western ideals, which are inevitably seen as associated with racism’ [47, p. 769–770].
S T R A T E G I E S	
1. Solidarity-building Alternative governance systems: regional economic (trade) and political solidarity; regional/Third World blocs (e.g. UNASUR, G77, NAM)/Global South leadership Linguistic, cultural, and historical ties (e.g. Community of Portuguese Language Countries) Working class/ideological/political solidarity (e.g. ALBA) Building of domestic/regional capacity Countering imperialism; reducing dependency on the West	‘UNASUR was formed as contestation to the “morality” of neoliberal governance and hence regionalism has been about addressing political roots of the struggle for inclusive development and citizenship in the region’ [84, p. 670]. ‘For many Pakistanis, the Cuban experience was a revelation. In the first instance, there was a stark sense of disbelief that these individuals came to Pakistan of their own free will and that they stayed well beyond the point that most global relief efforts had wound down’ [48,para.10]. ‘The Cubans also distinguished themselves from the rest of the relief effort by … living in tents under the same conditions as those displaced by the earthquake … ’ [48, para. 11]. ‘Cuba’s assistance to African countries in the health sector’ is framed by its role ‘as a prominent member of the Non-Aligned Movement and the Group of 77, [in the context of which it has] persistently evoked the need for unity and cooperation among developing countries in their collaborative struggle against exploitation’ [56, p. 32].
2. Health justice approaches Social/health/human rights Health equity Addressing social determinants of health (SDOH); intersectoralism	‘ … Stimulating political participation; extending and defending human rights’ … ‘guaranteeing the institutional and intersectoral coordination required by program implementation’ [77, p. 177]. ‘Unlike the more traditional disease-specific approach, [Brazil focuses on] strengthening and transforming health systems as a whole … [Fiocruz cooperation] consider[s] not only the biomedical aspects of disease, but also the social and environmental determinants of health’ [49, p. 2131]. Cuba ‘now heeds a community-based approach, centered on prevention and health promotion, with personnel living in the community … making care much more attuned to local needs’ [50, p. 714–15].
3. Mutual exchange and collective justice Reciprocal exchange of resources for mutual benefit Shared agenda-setting Social debt/gratitude for supporting revolutionary movements and restitution/reparations	‘At the domestic level, internationalism, and especially its medical diplomacy, have provided the country [Cuba] with material capital including economic gains from contracts, credit, and trade, which have helped the island sort out some of its economic difficulties’ [93, p. 100–101]. ‘Both South-South cooperation and triangular cooperation represent an increasingly important dynamic of interchange in the Iberoamerican region with unique guiding principles of horizontality and consensus’ [51, p. 14]. ‘Moral obligation to provide restitution for [South Africa’s] destabilization policies pursued throughout the 1980s and which are responsible to a great extent for the deterioration of infrastructure in neighbouring countries’ [88, p. 929]. Quid pro quo: ‘China’s foreign-aid medical teams have successfully promoted South-South cooperation and played a positive role in promoting China’s entry into the UN, also safe-guarding national sovereignty in regard to the Taiwan issue’ [52, p. 92].
4. Challenging interests of dominant classes in the health arena Challenging power of professional elites Challenging domestic and global capital (e.g. pharmaceutical companies) Challenging privatization Democratizing global governance systems	Quoting a Venezuelan emergency room doctor on why Cuban cooperation was needed: ‘The doctors at the private clinics that serve the rich and middle class have no interest in serving the majority of the population’ [53, p. 74]. ‘IBSA members implemented strategies questioning prevailing international health arrangements that they considered unjust and inequitable. As a result they challenged the existing order and proposed an alternative model’ [54, p. 302]. ‘To strategically place South America in a stronger and unified position addressing health issues in global governance’ [6, p. 9].

## Results

### Descriptive findings

**Figure 1.**
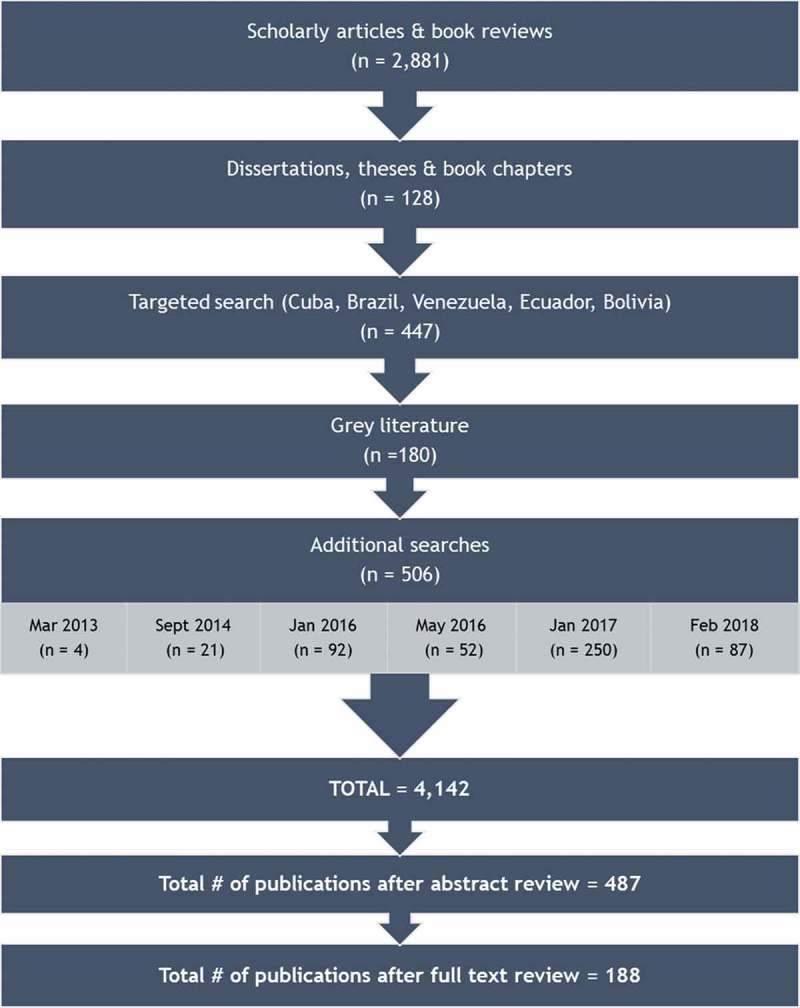
Flow chart of search process and publication selection.

Initially, 4,142 pieces published between 1992 and 2017 were captured by the search terms. Our abstract review yielded 487 publications, each of which then underwent a full-text review. In total, 188 publications met all inclusion criteria (Figure 1).

The original language of two-thirds of the literature is English, with the remaining third nearly equally divided between Spanish (18%) and Portuguese (16%). Three-quarters of the included publications are academic (including articles, books, and book chapters), with the remaining quarter grey literature.

**Figure 2.**
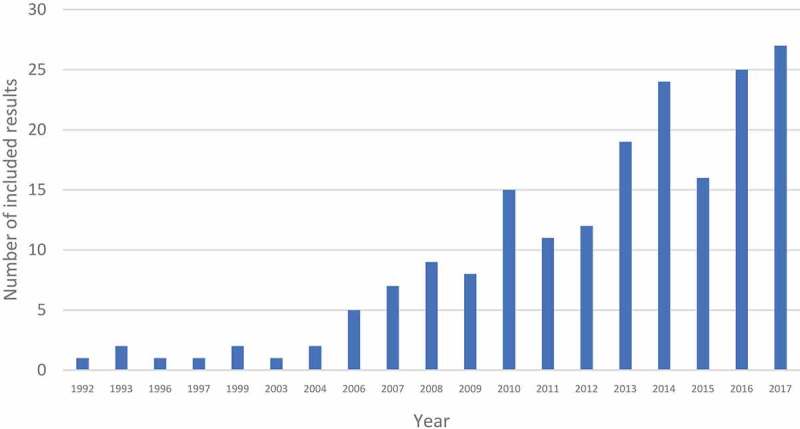
Number of SJSSC publications identified in scoping review, 1992–2017.

Figure 2 presents the evolution of publications on SJSSC since the early 1990s as identified in this review. The rate of publications increases circa 2006 and continues to rise to the present day. Several global developments could account for such trends. The rise to power of left-of-center and center-left governments in various Latin American countries (Brazil, Venezuela, Bolivia, Ecuador, Chile, Argentina, Uruguay) may have facilitated a new era of SJSSC in health and therefore an accompanying growth in the literature.

Much of the literature describes multiple types of cooperation, health activities, and social justice-oriented features. The most common type of SJSSC is bilateral cooperation, present in nearly three-quarters of the publications, followed by regional cooperation, which is described in just over a quarter of the publications. Regarding the content of the cooperation, we observed a great variety of activities where no single activity seems to dominate. Health-related training and education; pharmaceutical production and distribution; primary health care and human resources; and knowledge transfer and research are present in between one-third and one-half of the included publications. The social justice component of the collaboration is most commonly manifested via shared values and agenda-setting and responsiveness to local needs, followed by: equitable partnering; policy autonomy and goals; reciprocity; and restitution, reparations, and social debt.

When we categorize countries involved into ‘donors’ (major partners) and ‘recipients’ (junior partners), Cuba and Brazil emerge as the clear dominant ‘donor’ countries in the SJSSC literature. On a much smaller scale, China, India, South Africa, and Argentina also appear as ‘donor’ countries. Other ‘donor’ actors include Southern multilateral agencies, especially the IBSA (India, Brazil and South Africa) Dialogue Forum and BRICS (Brazil, Russia, India, China, and South Africa); regional organizations, particularly the Union of South American Nations (UNASUR), the Bolivarian Alternative for the Americas (ALBA), and the Pan American Health Organization (PAHO) – the latter typically involved as an intermediary in triangular cooperation agreements. The significance of this new expansion of SJSSC cannot be overemphasized and signals a departure from previous decades when the Southern ‘good Samaritan’ role in global public health was almost exclusively played by Cuba [34,35] and China. Among the ‘recipient’ regions, Africa is most frequently highlighted, then Latin America and the Caribbean. Among individual countries in Africa, Mozambique, Angola, Guinea-Bissau, São Tomé and Principe, and South Africa are the primary ‘recipients.’ In Latin America and the Caribbean, the main ‘recipients’ are Venezuela, Haiti, Bolivia, Brazil, and Ecuador.

Notably, Brazil appears as both a leading ‘donor’ – largely due to its health cooperation with Portuguese-speaking African countries – and ‘recipient’ because of the literature covering its Mais Médicos program involving a PAHO-mediated arrangement for recruiting thousands of mostly Cuban doctors to fill shortages in primary health care services to underserved areas in Brazil. [Note: After newly-elected President Bolsonaro and allies stepped up criticism of the quality of Cuban doctors, the ‘dangers’ of the political influence of the Cuban regime in Brazil, and the doctors’ shared pay arrangements, Cuba unilaterally pulled out of Mais Médicos. The Brazilian government’s promise that all positions would be filled by national physicians has not borne out: at writing, over 1,000 Brazilian doctors had resigned after just three months, contributing to over 2,000 vacancies across the country [36]. This situation particularly jeopardizes access to care in the poorest and most remote municipalities and in Indigenous areas [37].]

### Critical thematic analysis

In Table 3 we present the themes we identified in relation to SJSSC values and strategies, along with theme components and a selection of illustrative quotes from the literature.

#### Thematic analysis: values

**1. Anti-hegemonic world view** refers to anti-imperialist and anti-neoliberal revolutionary visions as well as internationalism and challenges to uneven North–South power. As argued by Carrillo Roa and Silva, under President Lula da Silva ‘ … Brazil promoted a multipolar international system, strengthening multilateralism as a means to reduce power asymmetries and promote the claims of developing countries’ [55, p. 6]. An anti-hegemonic world view is also exemplified by anti-imperialist sentiments, such as Cuba’s 'distinctive discourse evoking the essential commonality of poor, developing countries, sharing similar oppressive colonial legacies, with their development blocked by what Castro called “the unjust and obsolete international economic order prevailing in the world”’ [56, p. 32]. A more reformist variant is apparent in the BRICS’ explicit rejection of ‘many models used by Western donors’ [57, p. 16].

Anti-neoliberal views are yet another manifestation of this theme in the literature, as illustrated by Venezuela’s Barrio Adentro program as ‘ … a model of South-South international relations, where aspects of solidarity and complementation predominate, as opposed to the imposition and competitive characteristic of neoliberal health policies’ [58, p. 242]. These quotes (see Table 3 for more examples) highlight values associated with Marxist and/or socialist traditions both in revolutionary internationalist and social-democratic forms.

**2. Equity-oriented and redistributive political values (within national politics and translated into cooperative actions)** comprises: Socialism of the South, social democracy, social inclusion, and participatory democracy. Socialism of the South refers to the wave of elected left-wing governments that emerged in Latin America around the turn of the 21^st^ century reaching a peak circa 2012, and the interaction between the political values invoked in national political struggles and those imbued in cooperation efforts. Cuba, long pre-dating the SJSSC cooperation efforts of other Latin American countries, has been a pioneer of such approaches, as illustrated by De Vos et al.: ‘Cuba’s interventions are living proof of the viability of its socialist societal project, in which – even under difficult economic circumstances – health for all has become a reality … Sending doctors all over the world, Cuba not only addresses immediate humanitarian needs but also makes a statement that alternative development strategies are at hand and are even quite successful’ [59, p. 774].

Brazil’s role in the early 21^st^ century linking domestic values to international cooperative efforts is also stressed: ‘Since the “movement for health reform” succeeded in enshrining the right to health in the country’s 1988 Constitution, the development of the health system has been shaped by a powerful national health system “epistemic community” which has brought activists, academics, politicians and bureaucrats together to push for rights-based universal access to health care’ and is ‘very influential in key institutions involved in Brazilian health co-operation, including the Oswaldo Cruz Foundation (Fiocruz)’ [60, p. 2].

**3. Egalitarian terms of cooperation** includes elements such as horizontal interchange, solidarity-oriented humanitarianism, responsiveness to local needs, and context-relevant solutions. These elements are quintessentially seen in Cuba’s approach to ‘health in an egalitarian framework. The emphasis is not only on the doctor’s craft but also on increasing understanding of health determinants and prevention within communities’ [61, p. 90].

Chinese officials ‘summarize China’s attitude toward China-Africa relations [as]: “remaining faithful, valuing real results, cultivating kinship-like quality and being sincere”’ [62, p. 1]. Another element – responsiveness to local needs – is exemplified by how Cuba responds to needs prioritized by cooperant countries, including through ‘free provision of health professionals and technicians mainly directed towards primary health care in rural and remote areas in the spirit of altruism and solidarity’ [63, p. 384].

While many authors point to humanitarian values as being important to SJSSC, solidarity-oriented or altruistic humanitarianism differs markedly from the more typical charity-oriented activities of aid efforts responding to ecological disasters and wars. Instead, SJSSC humanitarianism involves transformative efforts, for example in triangular efforts supporting Haiti: ‘ALBA has been particularly active in the provision of health services, by financing Cuban medical brigades deployed to work in isolated communities in rural Haiti. The programme is highly regarded by recipient communities … the features that are often held up as the distinctive characteristics of ALBA cooperation in Haiti are the values of Bolivarian solidarity, genuine partnership and non-conditionality’ [64, p. 171–2].

**4. Reciprocity** covers harmonized policies, respect for national autonomy and sovereignty, and the notion of ubuntu. The latter refers to ‘African unity, interdependence and reciprocity, based on the perception that unity and the development of shared positions played a key role in prior achievements in health and in addressing economic and political determinants of health’ [65, p. 1].

While shared interests and respect for self-determination potentially overlap with values of egalitarian terms of cooperation and non-interference in domestic politics, reciprocity also merits separate consideration. A widely relevant articulation refers to Latin America: ‘Despite difficulties in defining, there is a consensus on its governing principles: solidarity and equal relationships, reciprocal benefits, respect for national sovereignty, shared responsibility, noninterference, nonintervention, self-determination, and independence’ [66, p. 369].

#### Thematic analysis: strategies

**1. Solidarity-building** incorporates both past and current approaches, such as historical efforts including the Non-Aligned Movement and the G77, and contemporary resistance to the Washington consensus around neoliberal capitalism and the exercise of leverage vis-à-vis corporate power. As observed, ‘the principles of Cuban health cooperation have always had an international dimension … to express solidarity with countries in need’ [67, p. 107].

Ideological and political ties also underscore ‘a sense of South-South solidarity: Brazil sees its overseas health connections as integral to a broader goal of strengthening relationships among countries within the global south to challenge what it views as the concentration of influence among the northern powers’ [68, p. 79]. Stated more pointedly, ‘The spirit of UNASUR … diverged from the US-dominated hemispheric mold of cooperation … as well as from post-Cold War initiatives inspired by neoliberal open-regionalism,’ instead forming ‘part of a struggle for post-hegemonic regionalism’ and to ‘counter the Washington Consensus’ [69, p. 262, p. 267].

In addition to discussion of regional economic/trade arrangements as challenging dominant global governance arrangements, the literature signaled SJSSC’s role as a means to enhance domestic capacity in health and reduce dependency on Northern/Western donors, viz.: Cuba’s efforts to address physician shortages in dozens of settings and Brazil’s explicit promotion of ‘national self-reliance and technological independence through its co-operation projects’ [70, p. 267].

The extent to which solidarity-building appeared in the literature was thus a conscious reflection of SJSSC’s intent to utilize collective blocs and inter-country support outside of the (Northern) nexus of power to exercise more socially just forms of health cooperation, manifesting the values (such as egalitarianism, inclusiveness, participatory and health justice/collective visions) articulated in the prior section. A prime example is Brazil’s approach, especially in relation to Portuguese-speaking countries: ‘The purpose is to go beyond traditional forms of international aid and to redefine Brazilian cooperation in health as “structural”, i.e. centred on strengthening recipient-country health systems institutionally, combining concrete interventions with local capacity building and knowledge generation, and promoting dialogue among actors, so that they can take the lead in health sector processes and promote formulation of a future health development agenda of their own’ [71, p. 103].

**2. Health justice approaches** yielded three inter-related dimensions analyzed around social and human rights, health equity, and addressing SDOH/intersectoralism. For example, Argentine international cooperation aims to ‘promote initiatives centered on social inclusion, sustainable development, solidarity between peoples, the defense of human rights and equity in all its forms’ [72, p. 7]. As well, UNASUR’s health cooperation efforts stress the importance of ‘evaluating policies around intersectoralism and social participation, health promotion and the reduction of inequities,’ in addition to incorporating the study of social determinants of health into the educational curriculum for health professionals [73, p. 2706]. Interestingly the International Labour Organization (ILO) has also noted that it relies heavily on SJSSC to advance the decent work agenda [74].

This theme also links domestic politics to SJSSC, not only as per the related value above, but in its actual implementation: for example, Cuba connects its constitutional guarantee of free universal health care as a basic human right and responsibility of the state to its role in the Barrio Adentro program in Venezuela. As such, ‘What began as the implementation of one of the core values of the revolution, namely health as a basic human right for all peoples, has continued as both an idealistic and a pragmatic pursuit’ [75, p. 100]. Similarly, ‘Brazil’s health diplomacy reflects the social democratic principles of its constitution and efforts to construct a universal health care system more than traditional foreign policy objectives’ [76, p. 70].

**3. Mutual exchange and collective justice** was perhaps the most inductive and semantically driven theme to emerge from this review. The components identified (see Table 3) drew from depictions of the crafting of shared agenda-setting, mutually beneficial results, and recognizing or rectifying a social debt. An unusual but telling finding was work that identified the theme of restitution in terms of post-apartheid South Africa seeking to compensate for the wrongs committed by the apartheid regime’s military forces across Southern Africa. More generally, SJSSC ‘counts on a relation between partners that offer and demand benefits amid conditions of reciprocity’ [77, p. 49], and as a kind of ‘collective action … that is simultaneously idealistic and pragmatic’ [78, p. 76].

The literature on Cuban SJSSC has documented its initial solidarity-oriented appreciation for countries that had supported Cuba’s revolution (both before and after 1959) or that were engaged in similar struggles. Yet over time, Cuba’s medical internationalism has extended well beyond countries with active anti-hegemonic movements [79].

Various authors emphasize Cuba’s current ‘dual goals of capitalizing on its highly educated population as a source of export income while pursuing its humanitarian goals of international solidarity’ [80, p. 45]. Some discussions of the Cuba–Venezuela collaboration go even further, underlining the ‘fostering [of] fraternal ties between their countries and peoples … trading mutually beneficial goods and services under agreed upon conditions; and … pursuing common political continental objectives under the banner of Bolivarianism with programs such as ALBA and others’ [81, p. 108]. Aspirationally and in practice, SJSSC strategies seek to ‘reach a true harmonization of interests, respecting the leadership of each partner … and benefiting from a real interchange among all and reciprocity in the efforts realized’ [82, p. 274–5].

**4. Challenging the interests of dominant classes in the health arena** and its four components visibly demonstrate the ways in which SJSSC harnesses social justice values via concrete actions rather than rhetorical devices. Challenging the power of professional elites transpires through the training of literally hundreds of thousands of health professionals (i.e. through the Latin American School of Medicine [ELAM] and other Cuban training efforts). It is also manifest in the ways that medical elites (especially those in private practice) in Brazil, Venezuela, and other settings assert their (ideological) opposition to the presence of Cuban doctors engaged in health cooperation due to the ‘implications for the earning potential of these professionals’ [83, p. 337].

Challenging domestic and global capital is evidenced in works outlining IBSA and South–South civil society solidarity against private pharmaceutical interests *and* the policy mechanisms utilized by organizations like UNASUR – such as regional government purchasing of medicines and taking a collective stance at the WHO and the World Trade Organization (WTO) to protest monopolistic pharmaceutical pricing practices – directly defying corporate power. For example, UNASUR’s health institute, the South American Institute of Government in Health (ISAGS), has sought to strengthen the position of member states vis-à-vis pharmaceutical companies and enable joint negotiation and purchasing strategies. UNASUR has also staked a position at the WHO on the ‘monopolist[ic] position of pharmaceutical companies on price setting and generics’ and pointed to the illegitimate role of Big Pharma in pushing the WHO to tackle counterfeit medicines that cut into their profits [84, p. 671].

Challenging privatization, while less directly articulated in the literature than the other two components, encompasses such activities as calling for universal public accessibility of antiretrovirals and advocating for publicly delivered and financed health care systems. In that sense, Cuban cooperation serves as a counterpoint to the ‘ … dominant neoliberal discourse that advocates privatization and profit-driven health services’ [59, p. 773].

In the final realm, UNASUR’s South American consensus around the need to democratize global health governance involves efforts to ‘advocate [for] more inclusive models of global health governance’ [6, p. 2] at the WHO in particular and to broadly ‘contest the existing order in the global governance of health’ [6, p. 1].

## Discussion

Our initial scoping review questions were concerned with identifying and characterizing the nature and features of SJSSC in the literature to rectify the unverified broad assertions made about SSC’s inherently more just and equal nature. To that end, we found a notable and dynamic presence of SJSSC in the literature. We gleaned the values underpinning SJSSC to comprise the following themes: anti-hegemonic world view; equity-oriented and redistributive political values; egalitarian terms of cooperation; and reciprocity. These themes were accompanied by a quartet of strategies represented that echo (but do not correspond exactly with) the values carried out through cooperation: solidarity-building; health justice approaches; mutual exchange and collective justice; and challenging interests of dominant classes in the health arena. While there was an inevitable thematic overlap in the quotes we highlighted (since we sought to avoid decontextualized ‘sound-bite’ quotes), we believe that each theme has clear distinguishing features.

### Finding bona fide SJSSC

Our identification of two sets of themes suggests that the principles, intentions, and goals (*values*) of SJSSC are not identical to the actual approaches (*strategies*) to cooperation, even as the latter seek to embody the former. This differentiation is an important means of enabling evaluation of the disconnect between much SSC discourse and its actual practice. Just one example, India’s Pan-African e-Network, demonstrates the need for this differentiation. The Indian government touts this project as a ‘non-hierarchical mutual[ly] beneficial partnership based on solidarity’ [85, p. 2], yet ‘the top-down, standalone and blueprint approach limits the agency and appropriation of the project’ [85, p. 15].

Although such assessments go beyond the scope of this review, our findings do have implications for policy and practice around bona fide SJSSC in terms of both values and the strategies undertaken. While a range of countries engage in SJSSC, the clearest finding is that Cuba is a leader in infusing SJSSC values into practice, such as its ‘beliefs that healthcare is a universal right for all people, regardless of who and where they are’ [86, p. 23]. Given difficult economic conditions, Cuba’s formerly gratis medical education programs and thousands of internationalist primary care physician cadres have now become the largest source of government revenue [79] (as per mutual exchange and collective justice strategies). Still, its equity-oriented and redistributive values (eschewing commodity extraction, worker exploitation, or invasion/occupation in its foreign policy) remain a hallmark of its approach: ‘although socialist ideology professes the goal of ameliorating the human condition, only Cuba has made health [cooperation] a defining characteristic of its “revolution”’ [87, p.1].

In addition, the Cuba–Venezuela case suggests that, even in the egalitarian context of a SJSSC exchange, the health ‘donor’ country can exercise political influence in the ‘recipient’ country (e.g. on the structure of the Venezuelan health system and foreign policy) [53,89].

Cuba remains, however, a unique case that resists classification. In particular, it is the only country achieving substantial political influence without accompanying economic or political power (i.e. appearing as a model of resistance against imperialism, alone and without resources; as an example of values-based foreign policy in African independence wars; and acting as a leader in foreign health policy, based on local needs).

This contrasts with broader claims made about SSC that have proliferated in development aid circles (e.g. at the 2011 Busan High-Level Forum [90, p. 197]) and mention vague characteristics without specifying concrete approaches to cooperation. Indeed, we hope that an important outcome of this review is to qualify the exuberance around SSC in mainstream global health diplomacy by stressing vibrant discussions of SJSSC as resisting asymmetrical aid dependence, advancing equity-oriented and redistributive policies, and countering imperialist approaches to health diplomacy. In this regard, the characterization of SJSSC we have elaborated could further animate sharing of SJSSC experiences and practices across different regions of the world (reminding readers that we are not providing a topology of all forms of cooperation).

### Understanding the politics of SJSSC

What distinguishes SJSSC’s health diplomacy in the literature identified in this review is that it is not simply another ‘soft power’ form of acquiring political goodwill and strategic advantage, but rather a conscious means of both countering mainstream (often US-dominated) global health agendas and utilizing the alternative of social justice-oriented cooperation as a form of ‘solidarity diplomacy’ [78]. This plays out in SJSSC’s conscious efforts towards ‘greater global justice on health worker migration, health financing or medicines access … [and] to challenge losses from health worker migration, to strengthen local production or negotiate fairer global measures on innovation and intellectual property’ [91, p. 15].

A prime illustration cited in the literature is Cuba’s utilization of SJSSC as a ‘buffer’ against the US’s decades-long economic embargo [92] and to counteract Cuba’s ‘international isolation’. As such, SJSSC ‘is one of the most outstanding mechanisms of rapprochement with developing countries from all over the world … and has acted as a protective shield in a permanently hostile environment, significantly contributing to the regime’s survival and legitimation on the world stage’ [93, p. 100–101].

Our understanding also seeks to withstand the narrowing of SJSSC into a series of technical constituent parts, for example its low cost: ‘the “comparative advantage” of South-South health aid, as indicated by the PRC [People’s Republic of China] and Cuban projects studied, may be in their capacity to provide, at very low cost, large numbers of health-care professionals’ [94, p.80]. Instead, in showing the distinct processes/strategies and substantive content of SJSSC, we open the door to further studies that show how SJSSC differs from other forms of development aid in health, with important implications for diverse health and development outcomes, including policy autonomy, health diplomacy, and alignment with health equity policies.

We further argue, unlike others [95], that technical cooperation cannot be dissociated from the larger context of power asymmetries between players or conscious efforts to minimize them. Much of the literature on SSC in biotechnology, for example, remains apolitical [96]. Nonetheless, deeply considering political framing does not mean that SJSSC is solely a reflection of the particular interests of political leaders. For this reason, while various key players in SJSSC were mentioned in our review (e.g. Castro, Lula), we sought to underemphasize the role of specific leaders; even as some instances of SJSSC have been personality-driven, at a conceptual level SJSSC indicates larger political considerations and struggles.

### A historic moment and historical trajectories

The scoping review revealed the centrality of Latin America (especially Brazil, Cuba, and UNASUR) in SJSSC, reflected in the major increase since 2000 in publications covering SJSSC and building on Cuba’s long-time leading role. Notwithstanding the emergence of BRICS and other LMIC actors, Cuba remains the most coherent and wide-ranging actor in SJSSC. Still, Latin America’s Pink Tide of leftist governments starting circa 2000 has been central to putting SJSSC on the wider scholarly map. Albeit accepting a pragmatic integration with the world economy, these governments became committed to domestic redistribution of revenues coming from the production surge/new discovery of commodities (and rising prices), rather than through equitable tax reforms. Fuller government coffers, often thanks to state-owned extractive enterprises, have also enabled heightened SSC and/or – when values expressed within national politics, including self-determination and responsiveness to local needs, are articulated in cooperative efforts – SJSSC.

Yet with shifting political winds starting in 2015, we may have already witnessed ‘peak SJSSC’. Indeed, between the time this scoping review of SJSSC was conceived in 2013 and its completion in 2018, Latin America’s Pink Tide became embattled, collapsing under right-wing governments in Brazil and Argentina, and facing ongoing turmoil and hostility in Venezuela (albeit with Mexico recently electing a left-wing government). Even with Cuba remaining as SJSSC’s standard bearer, these political changes have brought our review and the analytic themes it has engendered into sharper relief, providing more distance and new insights on the rhetoric and realities of SJSSC.

### Limitations, key issues, and future research possibilities

While this review could not quantify SJSSC’s representativeness in the overall practice of SSC, a preliminary scan of the literature revealed that almost 40% of relevant SSC articles fall under our definition of SJSSC. This suggests that what we characterize as SJSSC is among the most researched and discussed dimensions of SSC and further underscores the need for this scoping review.

This review encompasses government-to-government (or in certain cases regional [government] organization-to-government) cooperation; it does not cover the range of global health activities carried out by philanthropies, private companies, consulting firms, religious groups, universities, nongovernmental organizations, or social movements, which themselves comprise many forms of cooperation, only some of which are social justice-oriented [97].

Even as we made extensive efforts to capture grey and non-indexed literature, obstacles faced by many researchers in the Global South (especially in Sub-Saharan Africa) to publishing in academic and other widely-read venues meant that we undoubtedly missed some relevant SJSSC experiences.

We reviewed materials published from 1992 to 2017, corresponding with the modern era of global health and the quarter-century following the Cold War. We recognize that neither SSC nor SJSSC are new phenomena [66,98,99]; this review includes pieces covering historical accounts of SSC dating back to the height of decolonization in the 1950s and 1960s and the emergence of the Non-Aligned Movement (NAM) [100], even as the publication time frame of the literature we review emphasizes the growth of this arena in recent decades.

To note, this review did not historicize understandings of SJSSC, an important area for future research given shifts over time in the concepts and practices in this field. A key dimension is how class relations and class conflict within countries play out in understanding the nature of solidarity and its vicissitudes. For example, while Cuba’s SJSSC is clearly portrayed as a translation of socialist values of equity and redistribution at home into cooperation abroad, it has also engendered dilemmas around transnational solidarity when this coincides with shortages of medical materials and physician personnel domestically [101].

Moreover, our review was not designed to assess the quality of the evidence about SJSSC on the ground. Future research on how SJSSC is implemented and how its values, strategies, and everyday practices differ from SSC or other forms of cooperation (including North–South social justice variants) would be a valuable counterpoint to the present study.

This review also did not aim to capture the contradictions between SJSSC practices and larger foreign policy aims and commercial activities, again a crucial issue but beyond the framing of this kind of study. A prime instantiation is Brazil’s structural cooperation approach, particularly in Portuguese-speaking countries in Sub-Saharan Africa [82]. While Brazil’s cooperation contrasts with dominant forms of aid by foregrounding the agenda and needs of LMICs and, like China’s, in rejecting conditionalities, both of these countries’ extensive investments in African mining, construction, and other industries conflict with SJSSC values and strategies [102,103]. Brazil, China, and South Africa, among other extractive economies, are also at potential danger of reproducing North–South relationships (sub-imperialism) due to their regional, military, political, and economic weight [104].

Future research might examine such political contradictions between ideology and action in SJSSC in greater detail [105]. One relevant example is the reaction of local physicians’ organizations to Cuban cooperation missions. Another avenue for future studies is the role of powerful political actors such as Hugo Chávez or Fidel Castro in the development of contemporary SJSSC and the relation between economic exchanges and the class solidarity ideology that underpins most SJSSC. Lastly, further analytic attention might be focused on the contradictions between market-oriented and class-solidarity cooperation, as in the case of the involvement of Brazil, China, and South Africa.

## Conclusion

This scoping and critical literature review responds to the concern that much writing on SSC in health falls into little-grounded generalizations about the nature of cooperation carried out by former colonies and aid recipients in the Global South. Specifically, we argue, the presumption that SSC is necessarily more fair and respectful merits rigorous analysis. As such, we examined a quarter century of academic and grey literature, plus official government sources, for coverage of SSC with social justice-oriented features, drawing from an initial suite of ideas (even terms of engagement; anti-hegemonic stance; redistributive, radically democratic, equitable aims; and responsiveness to community needs) theorized from heterodox traditions including proletarian internationalism. We critically reviewed the materials identified to glean the predominant features of SJSSC expressed, especially in the context of Latin America and Africa, and iteratively employed thematic analysis to further refine our conceptualization of SJSSC based on how these ideas and practices were conveyed in the literature.

We identified 188 pieces that engaged with SJSSC ideas, the large number deriving from the generous time frame considered (spanning 1992 through 2017), the inclusion of grey literature and official sources, and the multiple languages covered. Based on full-text analysis along multiple axes, we found that our tentative definition needed elaboration to distinguish between the *values* conveyed as underpinning cooperation, and the particular *strategies* employed (reflecting the realization of these values). Our thematic analysis inductively identified four themes under each of these categories, yielding more detailed components.

This review uncovered notable articulation of a set of values and strategies that characterize SJSSC and bear relevance for the conceptualization, policy development, and practice of equitable health cooperation, demonstrating that anti-hegemonic health solidarity is possible even amid considerable political constraints. Whether the recent surge of SJSSC survives the end of the commodity boom and the mounting reversal of leftist (health) politics are matters of considerable concern for advocates of health justice everywhere.

Note: All source translations were conducted by the authors of this manuscript.

## Paper context

While South–South health cooperation is often portrayed as less asymmetrical than North–South aid, this has not been systematically studied. Our scoping review posits the existence of social justice-oriented South–South cooperation (SJSSC), characterized as counter-hegemonic, solidarity-oriented, and egalitarian. Our critical examination of the literature identifies a set of values and strategies that differentiate SJSSC from other forms of health aid. These findings are relevant for policy development and practice around equitable health cooperation.

## Dedication

This article is dedicated to the memory of the brilliant and committed Zabia Afzal, who left us far too soon …

## Appendix 1. Search terms

**Table:**

Main Categories	Search Terms
(1) Concept: Health/Well-being	(health* OR medic* OR biomedic* OR social welfare OR human welfare OR well-being OR wellbeing OR disease* OR illness* OR pharma* OR injur* OR disabilit* OR disabl* OR primary care OR drug* OR biotech* OR mortality OR morbidity OR vaccin* OR epidemiolog* OR scientific)
(2) Relationship Descriptors: Set 1	((north-south not (north-south gradient* OR north-south cline OR north-south axis OR north-south orient* OR north-south direction OR north-south line OR north-south split OR north-south transect)) OR (south-north) OR (south-south) OR (north-south-south)
(3) Relationship Descriptors: Set 2	(cooperation OR co-operation OR collaborat* OR regional coordination OR regional integration OR agreement* OR alliance* OR diplomacy OR linkage* OR pact*)
(4) Relationship Descriptors: Set 3	((joint AND (development OR production OR research OR planning OR program* OR initiative* OR effort* OR project* OR commitment* OR action* OR activit* OR venture* OR practice* OR process*)) OR joining forces OR ((mutual* OR common* OR shar* OR similar) AND (issue* OR problem* OR experience* OR duty OR responsibilit* OR strateg* OR priorit* OR agenda* OR goal* OR position* OR interest* OR benefi* OR advantage* OR reward* OR solution* OR challenge*)) OR common ground OR mutuality OR bi-directional OR symmetr* OR complementary development OR collective action* OR collective decision-making OR consensus)
(5) Political Descriptors	(egalitarian OR equit* OR equality OR ethical OR participatory OR independence OR self-relian* OR self-sufficien* OR self-sustain* OR reciprocity OR interdependen* OR ubuntu OR liberat* OR emancipat* OR empower* OR grassroot* OR decoloniz* OR bottom-up OR (human ADJ2 rights) OR (right ADJ2 health*) OR rights-based OR progressive OR non-interference OR solidarity OR self-determin* OR sovereign* OR sustainable development OR alternative development OR nondependen* OR non-dependen* OR (reduc* and (dependenc* OR poverty OR inequit* OR inequalit*)) OR autonom* OR people-orient* OR community-orient* OR anti-imperialis* OR anti-colonial* OR anti-capitalis* OR non-imperialis* OR non-capitalis* OR justice OR democra* OR social movement* OR horizontal OR indigenous OR southern-led OR southern-based OR southern perspective OR southern-generated OR African-driven OR African-led OR political commitment OR political will OR locally-determined OR social medicine OR (popular and (resistance OR participation OR forces)) OR inclusive OR social inclusion OR accessiblit* OR accessible OR political change OR social change OR non-Western OR subaltern OR reflexive OR locally governed OR regionally driven OR region-cent* OR socialis* OR feminis* OR anti-racis* OR altruis* OR accountab* OR transformative OR legitima* OR revolutionary OR decentrali* OR non-hierarchical OR counter-hegemon* OR mobiliz* OR regional power OR leftist OR left-wing OR activis* OR radical* OR regionali* OR internationali* OR redistributi* OR anti-globalization OR alter-globalization OR anti-US OR anti-america* OR anti-oppress* OR nationali* OR local governance OR regional governance OR fairness OR ((fair OR true OR real OR genuine* OR respectful*) AND (partnership* OR collaborat* OR cooperation* OR agreement* OR trade OR exchange*))
(6) Geographical Descriptors	(latin america* OR central america* OR south* america* OR southern cone OR Caribbean OR Africa* OR intra-africa* OR inter-africa* OR cuba* OR el salvador* OR brazil* OR venezuela* OR zimbabwe* OR kenya* OR tanzania* OR mozambique OR angola* OR namibia* OR Malawi* OR Zambia* OR Botswana OR Congo* OR Uganda* OR Swaziland* OR Lesotho* OR third world OR global south OR non-aligned countries OR non-aligned movement OR NAM countries OR developing countries OR low-income countries OR middle-income countries OR LMICs OR g-77 OR g77 OR group of 77 OR underdeveloped countries OR under-developed countries OR (southern ADJ3 countries) OR emerging economies OR resource-poor countries OR resource-poor settings OR resource-limited settings OR peripheral countries OR anchor countries)
(7) Organizations/Institutions	(barrio adentro OR global policy forum OR alliance for financial inclusion OR BRICS OR UNASUR OR southern african development community OR east african community OR Bolivarian Alliance for the Peoples of Our America OR ecos familiares OR equinet OR seatini OR latin american school of medicine OR IBSA OR COMESA OR CODESRIA OR SADC OR AU ECSA health community OR ALAMES OR People’s health movement OR World Social Forum)
(8) Scope of Cooperation	(transnational OR cross border* OR cross-national OR cross-countr* OR across national boundaries OR across ADJ2 borders OR multilateral OR trilateral OR foreign polic* OR regional OR neighbo$ring countr* OR bordering countr* OR inter-government* OR beyond borders)
(9) Substantive Area	((health* workers OR human resources OR personnel OR experts OR professionals OR providers OR practitioners OR caregivers OR physicians OR clinicians OR doctors) AND (migrat* OR train* OR retain* OR mentor* OR educat* OR teach* OR export* OR deploy* OR recruit* OR exchang*)) OR ((transfer* OR flow* OR exchang* OR shar* OR trade OR trading OR pooling) AND (technolog* OR knowledge* OR data OR information OR expertise OR skills OR know-how OR resources OR fund* OR services)) OR ((build* OR improv* OR enhanc* OR strengthen* OR develop* OR foster* OR promot* OR support* OR invest*) AND (research capacit* OR technical capacit* OR institutional capacit* OR research capabilit* OR indigenous capacit* OR local capacit* OR national capacit* OR domestic capacity* OR technical capabilit* OR technical knowledge OR technical skills OR technical competence OR institutional capabilit* OR infrastructure OR competence OR primary care OR primary health* OR health* services OR health* system)) OR capacity-building OR institution-building OR capacity-strengthening OR management-strengthening OR shar* research facilities OR mutual learning OR development* assistance OR technical assistance OR technical support OR development* aid OR foreign aid OR economic assistance OR financial support OR financial assistance OR knowledge diffusion OR human resource development OR institutional development OR national strategy OR health* advocacy OR (research ADJ2 development))

## Appendix 2. List of websites

1. African Leaders Malaria Alliance (ALMA): http://www.alma2015.org/
2. BRICS Policy Center: http://www.bricspolicycenter.org
3. Community of Latin American and Caribbean States (CELAC): http://www.celac.gob.ve/
4. Coordinadora Regional de Investigaciones Economicas y Sociales: http://www.cries.org/
5. Economic Commission for Latin America and the Caribbean (ECLAC): http://www.cepal.org
6. Codesria: http://newebsite.codesria.org/#&panel1-1
7. East African Community (EAC): http://www.eac.int/
8. East, Central and Southern African (ECSA) Health Community: http://www.ecsahc.org/
9. ELDIS: http://www.eldis.org/
10. Equinet- The Network on Equity in Health in Southern Africa: http://www.equinetafrica.org/
11. Health Policy Monitor: http://hpm.org/
12. ILO: www.ilo.org
13. The Inter-American Centre for Knowledge Development in Vocational Training (ILO/Cinterfor): http://www.oitcinterfor.org/en
14. Inter-American Development Bank (IDB): http://www.iadb.org/en
15. International Policy Centre for inclusive Growth | IPC: www.ipc-undp.org/
16. South American Institute of Government in Health (ISAGS): http://www.isags-unasur.org/index.php?lg=3
17. Japan International Cooperation Agency (JICA): http://www.jica.go.jp/english/
18. The New Partnership for Africa’s Development (NEPAD): http://www.nepad.org/
19. Pan American Health Organization (PAHO): http://www.paho.org/
20. Pan-African E-Network Project: http://www.panafricanenetwork.com/
21. People’s Health Movement (PHM): http://www.phmovement.org/
22. Programa Iberoamericano para el Fortalecimiento de la Cooperación Sur-Sur (PIFCSS): http://cooperacionsursur.org/es/
23. Research 4 Development: http://r4d.dfid.gov.uk/
24. Southern African Development Community (SADC): http://www.sadc.int/
25. SEATINI: http://seatini.org.zw/
26. SELA: http://www.sela.org/view/index.asp?ms=258&pageMs=26461
27. Shanghai Cooperation Organisation: http://eng.sectsco.org/
28. Smart Global Health: http://www.smartglobalhealth.org/
29. The South Center: http://www.southcentre.int/
30. The South-South Opportunity: http://www.southsouth.info/
31. Third World Network: http://www.twn.my/
32. UNEP South-South Cooperation: http://www.unep.org/south-south-cooperation/
33. United Nations Economic Commission for Africa (UNECA): http://www.uneca.org/
34. United Nations Office for South-South Cooperation: https://www.unsouthsouth.org/
35. The World Bank: http://www.worldbank.org/

### Government websites

1. Cuba: http://www.minrex.gob.cu/es/paginas-especiales/cooperacion
2. El Salvador: https://rree.gob.sv/
3. Ecuador: http://www.cooperacioninternacional.gob.ec
4. Venezuela: http://www.mppre.gob.ve/
5. Brazil: http://www.abc.gov.br
6. Argentina: http://www.cooperacionarg.gob.ar
7. Uruguay: http://www.auci.gub.uy/
8. Chile: http://www.agci.cl/
